# The Feasibility of 3D Printing Technology on the Treatment of Pilon Fracture and Its Effect on Doctor-Patient Communication

**DOI:** 10.1155/2018/8054698

**Published:** 2018-01-18

**Authors:** Wenhao Zheng, Chunhui Chen, Chuanxu Zhang, Zhenyu Tao, Leyi Cai

**Affiliations:** Department of Orthopaedic Surgery, The Second Affiliated Hospital and Yuying Children's Hospital of Wenzhou Medical University, Wenzhou 325000, China

## Abstract

**Purpose:**

The aim of this study was to assess the feasibility and effectiveness of the three-dimensional (3D) printing technology in the treatment of Pilon fractures.

**Methods:**

100 patients with Pilon fractures from March 2013 to December 2016 were enrolled in our study. They were divided randomly into 3D printing group (*n* = 50) and conventional group (*n* = 50). The 3D models were used to simulate the surgery and carry out the surgery according to plan in 3D printing group. Operation time, blood loss, fluoroscopy times, fracture union time, and fracture reduction as well as functional outcomes including VAS and AOFAS score and complications were recorded. To examine the feasibility of this approach, we invited surgeons and patients to complete questionnaires.

**Results:**

3D printing group showed significantly shorter operation time, less blood loss volume and fluoroscopy times, higher rate of anatomic reduction and rate of excellent and good outcome than conventional group (*P* < 0.001, *P* < 0.001, *P* < 0.001, *P* = 0.040, and *P* = 0.029, resp.). However, no significant difference was observed in complications between the two groups (*P* = 0.510). Furthermore, the questionnaire suggested that both surgeons and patients got high scores of overall satisfaction with the use of 3D printing models.

**Conclusion:**

Our study indicated that the use of 3D printing technology to treat Pilon fractures in clinical practice is feasible.

## 1. Introduction

Tibia Pilon fractures are complex injuries constituting 1% of all lower-extremity fractures and 5% to 10% of tibia fractures [1]. These fractures often result from high-energy trauma leading to multiple metaphyseal fragments, bone loss, displaced intra-articular comminution, and severe soft tissue injuries, which makes them one of the most difficult fractures to treat [2]. The fibula is usually fractured in these high-energy injuries. However, the treatment of Pilon fractures is a matter of controversy. The aim of operative treatment is anatomic reduction of the articular fragments and restoration of the distal tibial alignment while avoiding additional soft tissue trauma [3]. Most surgeons have recommended immediate open reduction and internal fixation (ORIF) after injury, because it can achieve anatomical reconstruction of the articular surface and satisfying functional outcomes postoperatively while sparing the soft tissue [4, 5]. Nevertheless, various complications, including soft tissue problems, delayed union, nonunion, malunion, implant failure, joint stiffness, and posttraumatic arthritis, may easily arise after surgery if not treated properly, which results in severe pain and affects the motor functions of the patients [6, 7].

In order to achieve better outcomes, we need more clinical exploration. ORIF need to be more accurate and individualized. Three-dimensional (3D) printing is a rapid prototyping technology that uses a 3D digital model to physically build an object in layer. In recent years, the use of 3D printing has allowed for the rapid manufacturing of custom-designed implants for orthopedics and reconstructive surgery, which can assist in accurate preoperative planning, as well as surgical strategy simulation and enhance communication with patients [8–10].

In this study, we compared the traditional surgery with surgery assisted by 3D printing technology in the treatment of Pilon fractures and ifs feasibility, efficacy, and safety will be included. Moreover, the communicative effectiveness of 3D printing between doctors and patients will also be investigated.

## 2. Materials and Methods

### 2.1. Patients

From March 2013 to December 2016, 100 patients with Pilon fractures were enrolled in this study. The inclusion criteria were as follows: (1) age older than 18 years old; (1) fresh closed fractures (within two weeks from injury), (2) unilateral Pilon fractures, (3) the contralateral normal tibia should not have any fracture, deformity, or history of surgery, and (4) at least 12 months of follow-up. The exclusion criteria were as follows: (1) the contralateral tibia fractures and/or dislocation, (2) old and pathological fractures, (3) open fractures, (4) severe soft tissue injuries (AO closed soft tissue injury grades [11] IC4 and IC5), and (5) multiple fractures.

**Figure 1:**
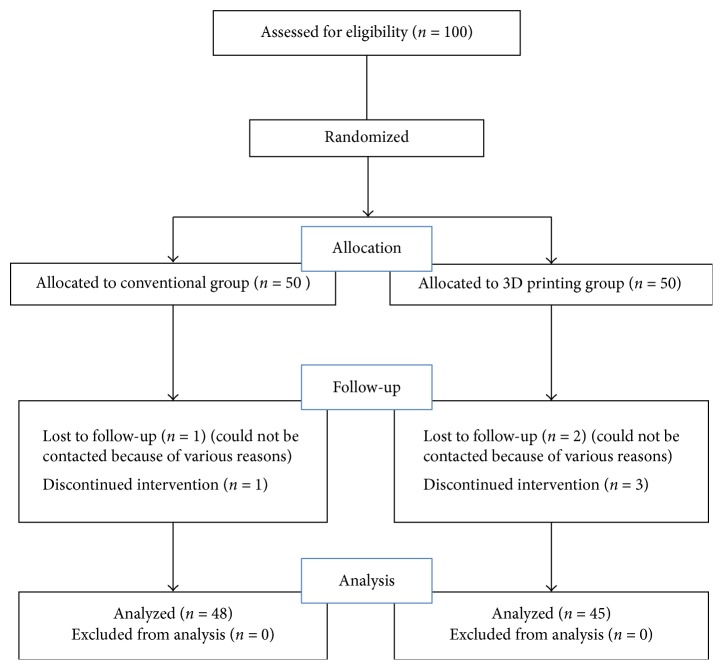
The flowchart of the study patients.

The fourth author of this study, who was not involved in clinical treatment, was responsible for assigning the patients to the two groups and subsequent data collection and statistics analysis. The 100 patients were randomized to conventional group (50 cases) and 3D printing group (50 cases) by random number table method. The flowchart of the study patients is presented in Figure 1. All the operations were performed by the same team. This study was approved by the Institutional Review Board of The Second Affiliated Hospital of Wenzhou Medical University.

### 2.2. Printing the 3D Model

We received CT scans of Pilon fracture patients from the Star PACS system (INFINITT, Seoul, South Korea) of our hospital. The original CT data were stored in DICOM format and 3-dimensional (3D) reconstructed using Mimics software v17.0 (Materialise, Leuven, Belgium), positioning by adjusting the threshold to reveal the intact structures of tibia and the bones around the ankle joint. The Region Growing command was used to separate the bones and soft tissues and establish the Mask of the tibia. The pixel set of the tibia was processed using the Calculate 3D form Mask command to produce the mirror image of the contralateral side, which was used as the 3D model of the injured side. The Mask pixel set of each fragment was established and the 3D Object was calculated using the Mask. The 3D model of the injured tibia was produced using Unite Boolean calculation and further processing for the noise reduction and smoothing of the tibia. The design data was then imported date into the 3D printing software (Cura Software v15.02) in STL format. After a 3D digital model was formed, we saved it in Gcode format and exported it to a 3D printer (3D ORTHO Waston Med, Inc., Changzhou, Jiangsu, China). Finally, the exact 1 : 1 models of the injured tibia and the mirrored contralateral tibia were fabricated.

### 2.3. Surgery Simulation

Surgeons were able to simulate the operation in vitro though the fracture model and the mirror model of normal side tibia. The structural feature of fracture was clear in the 3D printing models, and surgeons could mimic the intraoperative reduction and fixation maneuver accurately on the models. Moreover, we could choose suitable metal plates and screws in the real-size tibia model. The ideal length, location, and orientation were placed on the model. Then, the X-ray of the model would be taken for checking the proper position of the plate and the screws, which would be sterilized and stored for later use in surgery. In addition, the 3D printed model can be used intraoperatively as a reference for anatomical reduction of the fracture.

### 2.4. Surgical Methods

All the surgical procedure was performed by the same team as described previously [11]. The patients were subject to epidural anesthesia at the affected site and placed into the supine position on a standard radiolucent bed. Pneumatic tourniquet was applied to occlude the blood circulation. A broader approach to achieve satisfactory reduction was used as needed according to the fracture pattern. The skin and subcutaneous tissues were opened layer by layer. The tibial Pilon fracture was exposed clearly and the periosteum was appropriately stripped. After the elimination of soft tissues on the fracture bone, anatomical reduction of the fracture was made under direct vision. Before reduction, sufficient traction was used to maintain the reduction, and the obtained image was compared with radiographs of the contralateral ankle to ensure the reconstruction of the distal tibiofibular surface. The fracture fragments were fixed with Kirschner wires temporarily after the reduction. In 3D printing group, the preselected and prefabricated plate and screws determined by the 3D-printed model simulation were placed to fix fracture. However, in conventional group, the selection of plate and screws were only determined by the measurement during the surgery. The reduction was evaluated by the intraoperative C-arm X-ray machine. Finally, the incision was closed as usual.

### 2.5. Postoperative Management

The postoperative management was the same for all patients. The antibiotics were used within 3 days postoperatively. After operation, pressure dressing was applied on the wound surface and the affected side was raised to relieve the swelling around the wound. A postoperative X-ray of the affected tibia was taken the day after surgery. All patients remained strict toe-touch weight-bearing immediately postoperatively for approximately 3 months. A graduated physical therapy protocol was initiated on postoperative day 1 with crutches or a walker. The protocol was directed at gait training, active and passive lower-extremity range of motion exercises, and endurance training. Cast immobilization was used until wound healing, followed by immobilization with a removable cast-type boot. At approximately 3 months, weight-bearing was advanced as tolerated with supervision of physical therapy. This was followed by weaning of assistive devices for gait and the initiation of proprioception and endurance activities. The functional outcome of ankle was evaluated by the American Orthopaedic Foot and Ankle Society (AOFAS) hindfoot scores and visual analog scale (VAS) pain scores. The VAS pain score was used to measure the amount of pain patients felt between 0 and 10 points and contained word descriptors. The 100-point AOFAS scoring system considers a score of ≥90 points as excellent, 80–89 points as good, and 70–79 points as fair and a score of ≤69 points as poor [12].

### 2.6. Assessment Parameters

The operation time, blood loss volume, times of fluoroscopy during the surgery, and fracture union time were recorded. The fracture reduction was assessed on orthogonal simple radiographs and graded according to the score described by Burwell and Charnley [13]. The functional outcomes of ankle were evaluated by the range of ankle motion (dorsiflexion and plantarflexion), AOFAS, and VAS scores. Furthermore, the complications of the two groups were also evaluated in our study. The fracture healing was assessed radiographically through callus formation. We considered a healing time of less than 6 months as normal, between 6 and 9 months as a delayed union, and more than 9 months as nonunion [14]. Malunion was defined as more than 5 degrees of angular or rotational deformity [15]. Posttraumatic arthritis was described as painful range of motion with radiographic evidence of a narrowed joint space.

### 2.7. Questionnaire

A questionnaire was designed to allow doctors to evaluate the models. The details of the questionnaire were shown in Table 4. Ten orthopaedic surgeons evaluated the verisimilitude and effectiveness of the life-sized 3D model using the questionnaire. Besides, a questionnaire was also designed for patients and nonmedical professionals. This questionnaire was used to assess patient satisfaction with the levels of preoperative communication between the two groups. The details of the questionnaire were shown in Table 5. The scores ranged from 1 to 10 points, 1 point indicated that the model was useless/very poor/not realistic at all, and 10 points indicated that the model was very useful/very good/very realistic.

### 2.8. Statistical Analysis

Data were analyzed using Student's *t* unpaired test and the chi-squared test. *P* < 0.05 was considered statistically significant. Data are given as mean ± standard deviation. Statistical analyses were performed using SPSS version 18.0 software.

## 3. Results

### 3.1. Patient Characteristics

Of the admitted patients, a total of 7 patients (2 patients in conventional group and 5 patients in 3D printing group) were lost to follow-up because of various reasons. As a result, there were 48 patients remaining in the conventional group and 45 patients in the 3D printing group. There were 31 males and 17 females in conventional group and 35 males and 10 females in 3D printing group. Average age for patients in conventional group was 42.5 ± 9.0 years and in 3D printing group was 41.2 ± 9.3 years. In conventional group, the fracture was on the right in 28 patients and the left in 20. In 3D printing group, the fracture was on the right in 22 patients and the left in 23. According to the AO fracture classification [16], conventional group had 8 patients with type 43-C1 fracture, 17 patients with type 43-C2 fracture, and 23 patients with type 43-C3 fracture, whereas 3D printing group had 5 patients with type 43-C1 fracture, 14 patients with type 43-C2 fracture, and 26 patients with type 43-C3 fracture. Based on the AO soft tissue injury grading, there were 25 patients with grade IC 1, 16 patients with grade IC 2, and 7 patients with grade IC 3 in conventional group. And 28 patients with grade IC 1, 12 patients with grade IC 2, and 5 patients with grade IC 3 were included in 3D printing group. The most common cause of injury was fall from a height in both groups (27/48 versus 21/45). There were 38 patients with fibula fractures in conventional group and 33 patients with fibula fracture in 3D printing group. Time from injury to surgery of conventional group was 8.1 ± 2.3 days and it was 7.6 ± 2.5 days in 3D printing group. However, the demographic characteristics such as age, gender, side of injury, fracture classification, soft tissue grading, cause of injury, and time from injury to surgery were similar between the two groups (*P* > 0.05; Table 6).

### 3.2. Clinical Data

The results from clinical data were shown in Table 1. The operation time in the 3D printing group was 74.1 ± 8.2 min, which was significantly shorter than the conventional group (90.2 ± 10.9 min, *P* < 0.001). There was statistical significance in the intraoperative blood loss volume between the 3D printing group (117.1 ± 20.7 ml) and the conventional group (159.8 ± 26.5 ml, *P* < 0.001). In addition, the 3D printing group had significantly times of fluoroscopy during the operation (7.6 ± 2.2) than the conventional group (11.0 ± 2.9, *P* < 0.001). Fracture union time was also observed in the patients of the two groups. There was no significant difference in fracture union time between the 3D printing group (5.0 ± 1.1 months) and the conventional group (5.3 ± 1.2 months, *P* = 0.314). According to Burwell and Charnley grading, 41 patients achieved anatomic reduction, 3 patients achieved fair reduction, and 1 patient achieved poor reduction in the 3D printing group, while 36 patients achieved anatomic reduction, 8 patients achieved fair reduction, and 4 patients achieved poor reduction in the conventional group. No significant difference was found in fracture reduction between the two groups (*P* = 0.116). However, 3D printing group exhibited significantly higher rate of anatomic reduction (91.1%) than conventional group (75%, *P* = 0.040).

### 3.3. Functional Outcomes

The follow-up of the patients at least was 12 months. There was no statistical significance in the follow-up time between the 3D printing group (20.5 ± 3.7 months) and the conventional group (19.9 ± 3.3 months, *P* = 0.362). Compared to the initial situation, we found the ankle function of the patients in both groups was improved. As shown in Table 2, the motion of ankle dorsiflexion was 15.1 ± 4.8° in the 3D printing group and for the conventional group was 14.2 ± 5.0°. The motion of ankle plantarflexion was 27.4 ± 8.5° in the 3D-printing group and for the conventional group was 25.9 ± 8.7°. The two groups showed no significant difference in the range of ankle motion (*P* > 0.05). Besides, the mean VAS score in the 3D printing group was 2.6 ± 0.9 and for the conventional group was 2.9 ± 1.2, which was not statistical different (*P* = 0.218). Furthermore, the mean AOFAS score of the 3D printing group was 87.4 ± 8.7, whereas that of the conventional group was 84.7 ± 9.0. No significant difference was noted in AOFAS score between the two groups (*P* = 0.149). Moreover, in the 3D printing group, AOFAS score was scored as excellent in 24 patients, good in 18 patients, fair in 2 patients, and poor in 1 patient. In the conventional group, AOFAS score was scored as excellent in 23 patients, good in 14 patients, fair in 8 patients, and poor in 3 patients. The 3D printing group exhibited a significantly higher rate of good and excellent functional outcome (93.3%) than the conventional group (77.1%, *P* = 0.029).

### 3.4. Complications

The complications are summarized in Table 3. The total complication rate of the 3D printing group and the conventional group was 15.6% (7/45) and 20.8% (10/48), which was not statistically different (*P* = 0.510). Three patients with superficial infection were found in the 3D printing group while 4 patients were found in the conventional group, which were all successfully treated with antibiotics and daily dressing. There were 2 patients with delayed union in the 3D printing group while 3 patients were found in the conventional group. However, all of the delayed union patients healed after undergoing reinforcing weight-bearing and functional exercises within 9 months after surgery. Besides, 1 patient in the 3D printing group and 1 patient in the conventional group had radiological evidence of malunion during the follow-up period. One patient in the 3D printing group and 2 patients in the conventional group had some evidence of posttraumatic arthritis. One patient was managed conservatively and remained under follow-up, with a possibility of requiring surgical intervention in the near future. The other 2 patients had undergone arthroscopic debridement of anterior osteophytes. No other complications such as nonunion and deep infection were found in any group.

### 3.5. Questionnaire

The questionnaire from doctors showed that the overall satisfaction and usefulness of the 3D printing models were high (Table 4). For doctors, 3D printing models could provide a visual, comprehensive vision of fracture displacement, which can make the preoperative plan much easier. On the other hand, patients and their family members gave relatively high satisfaction scores for the quality of preoperative communication when the 3D printed fracture model was introduced to help the surgeons explain the patients' medical condition (Table 5 and Figure 6).

### 3.6. Typical Case

**Figure 2:**
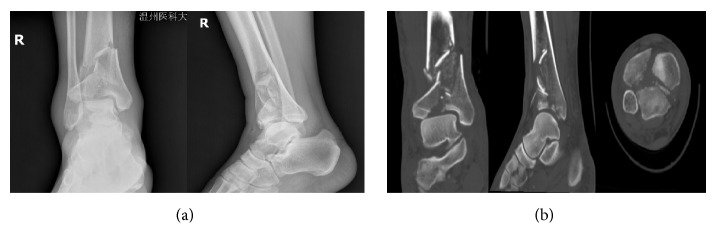
The patient's preoperative radiological characteristics of Pilon fracture. (a) The anteroposterior and lateral X-ray. (b) CT images of the fracture.

**Figure 3:**
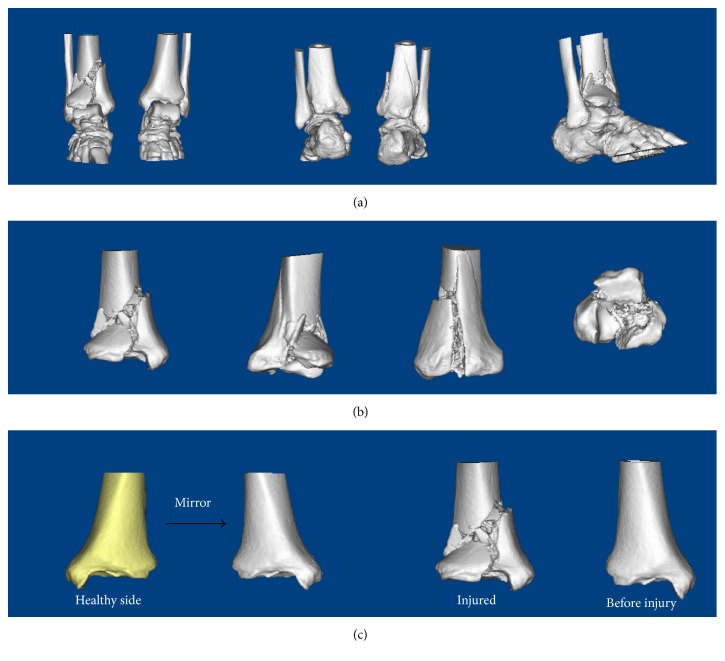
The 3D reconstruction and manipulation of the fracture in Mimics software v17.0. (a) The 3D reconstruction of Pilon fracture. (b) Using the CT bone segmentation function to separate the tibia. (c) Using the mirror imaging technique to reconstruct the model of tibia before injury.

**Figure 4:**
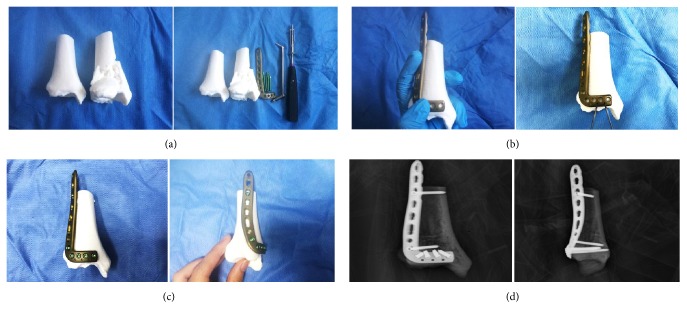
Simulation of the surgery in vitro. (a) Preparation of simulative surgery. (b-c) Simulating the surgery on the model. (d) The anteroposterior and lateral X-ray after the simulative surgery.

**Figure 5:**
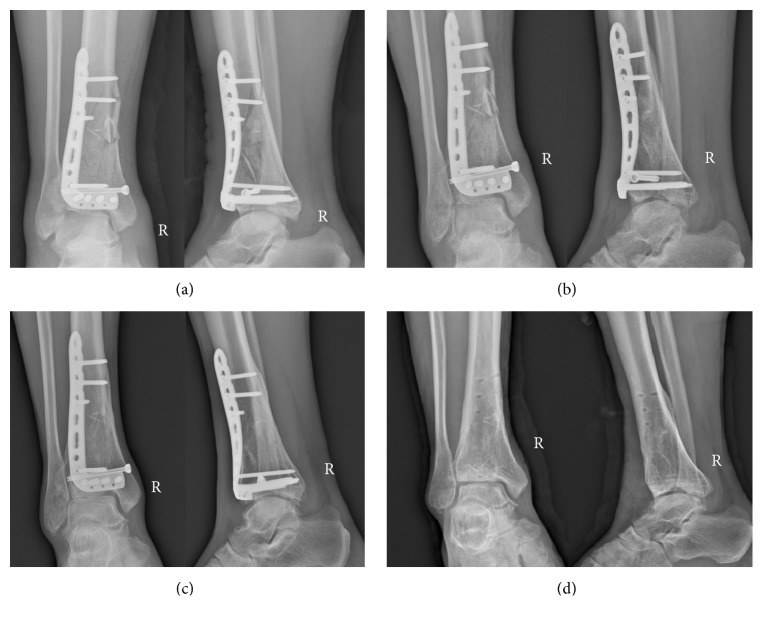
The radiography after the operation. (a) The anteroposterior and lateral X-ray after operation. (b) The anteroposterior and lateral X-ray at 3 months postoperatively. (c) The anteroposterior and lateral X-ray at 6 months postoperatively. (d) The anteroposterior and lateral X-ray at 18 months postoperatively (after internal fixation removal).

Male, 52 years old, falling from height, was selected as a typical case.  Figure 2 showed the preoperative X-ray and CT scan of fracture. Using the CT bone segmentation and mirror imaging technique in Mimics software v17.0, the characteristics of the injured tibia and the tibia before the injury were exhibited clearly (Figure 3). Afterwards, a 1 : 1 solid prototype of the fracture and the mirror model of normal side tibia were manufactured by 3D printing technology; then surgeons simulated the operation on the real-size mirror normal 3D printed tibia model. The outcome of the simulative operation was shown in Figure 4. Then the simulative operation was used to guide the actual operation. Postoperative review of X-ray film showed satisfactory fracture reduction and fixation (Figure 5). The plate and screw were in good position. This patient had a follow-up of 21 months. In this period, he did not suffer from any surgical complications and exhibited excellent AOFAS score.

## 4. Discussion

It is well known that the main goal of surgical treatment for Pilon fractures is to anatomically reduce and fix the intra-articular fragments and restore the length, alignment, and rotation of the distal tibia, which allows for earlier weight-bearing and mobilization [3, 17]. Therefore, a better overall preoperative understanding of the anatomical structure of the fracture is quite essential for the treatment. However, the X-ray and CT images could not provide a comprehensively understanding for the fracture, no more for the private custom.

In recent years, the application of the 3D printing applied in orthopedics was more and more common [18–20]. In this study, we found that 3D skeletal models can be printed according to patient CT data in combination with modern digital medical technology. Using 3D printed model, the fracture can be viewed in every direction to provide an accurate description of fracture characteristics. This enables the orthopedist to more effectively and comprehensively understand the specific details of the fracture, confirm the type of fracture, determine the displacement of the fracture line and the number of fracture fragments, examine the collapse and comminuting condition of the articular surface, verify the potential presence of bone defects, and determine whether bone graft is needed. Besides, 3D printing model is able to help orthopedist to make an individual, accurate, and reasonable surgical plan for patients. Surgeon can clearly and directly visualize the anatomical and pathological conditions of the fracture sites before surgery rather than having to begin operating with only partial knowledge. Therefore, the surgeon can accurately make surgical plans and simulate the surgical procedures before the procedure, which can highly increase the accuracy of reduction and the stability of the fixation [21]. The 3D printing also was used in ankle fractures and clavicular fractures, which showed its unique advantages, reducing the operation time, intraoperative blood loss, and the plasticity of plate [22, 23].

In the present study, we compared the conventional surgery with the surgery assisted by 3D printing technology in the treatment of Pilon fractures. In 3D printing group, we used the mirror technology to assist preoperative reduction, making the preoperative design, and simulate the operation. The size and location of the plate and screw could be made out. So it may account for the reduced operation time in the 3D printing group and may additionally help reduce intraoperative bleeding and fluoroscopy times leading to better functional recovery. Our results showed that the 3D printing group exhibited significantly shorter operation time, less intraoperative blood loss volume, and less times of intraoperative fluoroscopy as well as higher rate of anatomic reduction than the conventional group, suggesting that the suitable preoperative planning and individual treatment determined by 3D printed model could greatly optimize the surgical outcomes. In addition, this can reduce the exposure to radiation both for patients and for doctors and reduce the potential injury from surgery and anesthesia. However, the two groups did not differ significantly in functional outcome at the last follow-up period. As for the postoperative complication, there were no significant differences between 3D printing group and conventional group (15.6% and 20.8%). There is no obvious advantage of 3D printing technology for the prevention of complications.

**Figure 6:**
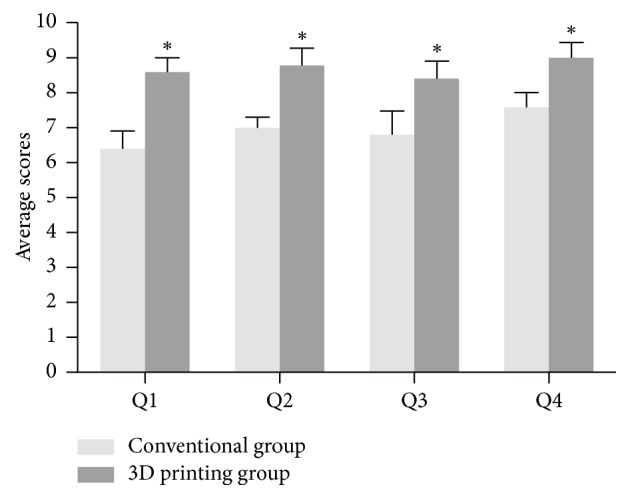
Survey questionnaire for patients and nonmedical professionals between the two groups. *∗*P<0.05 compared with conventional group.

The doctor-patient communication is very important in the clinic process [24]. It has been reported that 3D printed models could improve patient's understanding and compliance in orthopedic surgery [25, 26]. Fully understanding the fracture conditions can help patients and their family members coordinate surgical treatment with functional rehabilitation after surgery. In the current study, we made a questionnaire for both doctors and patients. According to Figure 6, conventional medical images are too complicated for patients and nonmedical professionals to fully understand, which increased the difficulty of doctor-patient communication. Our study found that the overall patient evaluation of the quality of doctor-patient communication was above 9 out of 10 points when the 3D fracture model was introduced to help explain the medical conditions and surgical plan to patients and their family members. Patients and their family members were thus satisfied with this type of communication, which effectively increased the ability of the patient or family member to understand the patient's medical condition while improving patient attitude toward and compliance with the doctor's recommendations [27]. On the other hand, doctors can also gain a better communication with both patients and their working team, which not only increases patient's understanding and compliance but also improves the performance and the collaboration of working team. Therefore, 3D printed model may be an innovative tool to improve the doctor-patient communication and achieve better clinical outcomes of the fractures.

Nonetheless, there are still some limitations for the 3D printing. Firstly, the 3D printing technology used in our study is only based on bone CT images which lack the information of the adjoining soft tissue and vasculature. Moreover, it will take a lot of preparation time for the model printing and simulated surgery; therefore it is not suitable for emergency cases. Also, 3D printing technology needs specific software, professionals, and 3D printers, which will undoubtedly increase human and financial expenses. Besides, this study enrolled only a small number of patients. To further confirm these results, high-quality randomized controlled trials with larger sample size were still needed.

Finally, in our study, 3D printing technology is still in the scientific research stage, not for charging, and our research funding supports it. What is more, the most work was done by our master graduate students. If this technology is promoted, more health resources will be needed.

## 5. Conclusion

In conclusion, 3D printing technology has provided orthopedic surgeons with powerful new tools and approaches. This technology is both safe and effective for the treatment of adults with Pilon fractures and has a significantly shorter operative time, less intraoperative blood loss, less times of fluoroscopy, and higher rate of anatomic reduction compared with the conventional group. Furthermore, 3D printing can help clinicians improve their theoretical knowledge and practical skills, reduce learning curves, eliminate common surgical complications, improve surgical quality, and provide a better communication between doctors and patients.

## Figures and Tables

**Table 1:** Comparison of the general conditions of the two groups.

	Conventional group	3D printing group	*t* or *χ*^2^	*P* value
*n*	48	45		
Gender				
Male	31	35	1.963	0.161
Female	17	10		
Age (year)	42.5 ± 9.0	41.2 ± 9.3	0.698	0.487
Cause of injury				
Fall from a height	27	21	0.856	0.652
Traffic accident	15	17		
Other causes	6	7		
Side of injury				
Left	20	23	0.833	0.361
Right	28	22		
AO/OTA classification				
43-C1	8	5	1.071	0.585
43-C2	17	14		
43-C3	23	26		
AO soft tissue grading				
IC 1	25	28	0.979	0.613
IC 2	16	12		
IC 3	7	5		
Associated fibula fracture (*n*)	38	33	0.438	0.508
Time from injury to operation (day)	8.1 ± 2.3	7.6 ± 2.5	1.062	0.291

**Table 2:** Comparison of clinical data between the two groups.

	Conventional group (*n* = 48)	3D printing group (*n* = 45)	*t* or *χ*^2^	*P* value
Operation time (min)	90.2 ± 10.9	74.1 ± 8.2	8.021	<0.001
Blood loss volume (ml)	159.8 ± 26.5	117.1 ± 20.7	8.625	<0.001
Times of fluoroscopy (*n*)	11.0 ± 2.9	7.6 ± 2.2	6.328	<0.001
Fracture union time (month)	5.3 ± 1.2	5.0 ± 1.1	1.013	0.314
Fracture reduction	—	—	4.305	0.116
Anatomic (*n*)	36	41	4.232	0.040
Fair (*n*)	8	3	2.227	0.136
Poor (*n*)	4	1	0.715	0.398^a^
Rate of anatomic reduction (%)	75%	91.1%	4.232	0.040

^a^ *P* value for continuity-corrected chi-squared test.

**Table 3:** Comparison of functional outcomes between the two groups.

	Conventional group (*n* = 48)	3D printing group (*n* = 45)	*t* or *χ*^2^	*P* value
Follow-up time (month)	19.9 ± 3.3	20.5 ± 3.7	0.916	0.362
Ankle dorsiflexion (°)	14.2 ± 5.0	15.1 ± 4.8	0.830	0.409
Ankle plantarflexion (°)	25.9 ± 8.7	27.4 ± 8.5	0.857	0.394
VAS score	2.9 ± 1.2	2.6 ± 0.9	1.239	0.218
AOFAS score	84.7 ± 9.0	87.4 ± 8.7	1.456	0.149
Excellent (*n*)	23	24	0.273	0.602
Good (*n*)	14	18	1.208	0.272
Fair (*n*)	8	2	2.454	0.117^a^
Poor (*n*)	3	1	0.198	0.656^a^
Rate of excellent and good outcome (%)	77.1%	93.3%	4.796	0.029

^a^ *P* value for continuity-corrected chi-squared test.

**Table 4:** Comparison of complications between the two groups.

Complications	Conventional group (*n* = 48)	3D printing group (*n* = 45)	*χ* ^2^	*P* value
Superficial infection	4 (8.3%)	3 (6.7%)	—	1.000^a^
Deep infection	0	0	—	—
Traumatic arthritis	2 (4.2%)	1 (2.2%)	—	1.000^a^
Delayed union	3 (6.3%)	2 (4.4%)	—	1.000^a^
Malunion	1 (2.1%)	1 (2.2%)	—	1.000^a^
Nonunion	0	0	—	—
Total	10 (20.8%)	7 (15.6%)	0.433	0.510

Values are expressed as number (%); ^a^*P* value for Fisher's exact test.

**Table 5:** Questionnaire for doctors.

Question	Subjective field	Average scores
(1)	Verisimilitude degree of the 3D printing model compared with the actual fracture	8.5 ± 1.0
(2)	Presentation of anatomical structure of fracture	8.4 ± 1.2
(3)	Usefulness of the 3D printing model for diagnosis and preoperative planning	8.3 ± 1.1
(4)	How much dose the 3D printing model help you to communicate with patients?	8.8 ± 1.0
(5)	Overall satisfaction with the 3D printing model	9.0 ± 1.1

**Table 6:** Questionnaire for patients.

Question	Subjective field
(1)	How much does the CT or 3D printing model help you to gain a better communication with doctors?
(2)	How much does the CT or 3D printing model help you to understand the surgical plan?
(3)	How much does the CT or 3D printing model help you to obtain a clear understanding of your condition?
(4)	Overall assessment of the conversation with CT or 3D printing model
